# Repeated Autologous Fat Grafting Significantly Increases Mastectomy Flap Thickness in Pre-Pectoral Multi-Stage Composite Expander-to-Implant Breast Reconstruction: Exploring the Concept of a Reverse Expansion

**DOI:** 10.3390/jcm14020337

**Published:** 2025-01-08

**Authors:** Lorenzo Costa, Andrea Weinzierl, Stefano Andreoli, Simone Schiaffino, Carola M. L. Catanese, Yves Harder

**Affiliations:** 1 Faculty of Biomedical Sciences, Università della Svizzera Italiana (USI), 6900 Lugano, Switzerland; costa.lorenzo95@gmail.com; 2 Department of Plastic, Reconstructive and Aesthetic Surgery, Ospedale Regionale di Lugano, Ente Ospedaliero Cantonale (EOC), 6900 Lugano, Switzerland; 3 Department of Plastic Surgery and Hand Surgery, University Hospital Zurich (USZ), 8091 Zurich, Switzerland; 4 Imaging Institute of Southern Switzerland, Ospedale Regionale di Lugano, Ente Ospedaliero Cantonale (EOC), 6900 Lugano, Switzerland; 5 Department of Plastic, Reconstructive and Aesthetic Surgery and Hand Surgery, Centre Hospitalier Universitaire Vaudois (CHUV), 1011 Lausanne, Switzerland; 6 Faculty of Biology and Medicine, University of Lausanne (UNIL), 1011 Lausanne, Switzerland

**Keywords:** composite breast reconstruction, repeated autologous fat grafting, pre-pectoral implant-based breast reconstruction (IBBR)

## Abstract

**Background/Objectives**: Combining autologous fat grafting with implant placement is meant to improve the quality of implant-based breast reconstruction. The present study explores the concept of multi-stage composite breast reconstruction with repeated sessions of autologous fat grafting to increase mastectomy flap thickness and provide better pre-pectoral implant coverage. **Methods**: Twenty-five consecutive patients underwent bilateral multi-stage composite expander-to-implant breast reconstruction and reverse expansion from August 2020 to April 2024. Subcutaneous thickness of the mastectomy flap was evaluated in predefined regions of interests of the breast on standardized MR images at two timepoints (before the first fat grafting session, with the tissue expander fully inflated, and 3 months after implant placement). Furthermore, the incidence of complications requiring surgery and implant-related complications were evaluated. All values are expressed as mean ± standard deviation, accepting statistical significance for a *p*-value < 0.05. **Results**: Patients underwent an average of 2.5 ± 0.6 fat grafting sessions, with a fat injection volume of 170 ± 60 mL per breast per session. The mean duration of the reconstructive process from mastectomy to final implant placement was 12 ± 5 months and the mean follow-up was 17 ± 8 months. The overall thickness of both breasts amounted to 190% of baseline thickness and was significantly higher in the upper breast quadrants than in the lower quadrants (*p* < 0.05). Tissue thickness increase correlated well with the number of fat grafting sessions and was independent of the patient’s weight gain. Complications requiring surgery occurred in eight breasts during the reconstruction, with iatrogenic expander puncture being the most frequent (three cases, 6%). During follow-up, only one implant-related complication was observed (one case of bilateral rippling, 4%). No breast animation or symptomatic capsular contracture were observed. **Conclusions**: Multi-stage pre-pectoral composite expander-to-implant breast reconstruction using autologous fat grafting is an effective concept for breast reconstruction. Despite the need for multiple surgeries, the significant increase in subcutaneous tissue thickness, resulting in better soft tissue coverage, compensates for the longer reconstructive process.

## 1. Introduction

Nowadays, two distinct approaches are generally offered for breast reconstruction: fully autologous breast reconstruction, ideally using microvascular flaps, or fully heterologous reconstruction, using breast implants. Autologous breast reconstruction is currently safely performed as a definitive solution and a durable alternative to implant-based breast reconstruction (IBBR), resulting in high patient satisfaction [1,2]. Due to the complex operative process, it must be performed by an experienced surgical team [3,4,5]. As this limits the availability of the technique, IBBR remains the most widely used method of breast reconstruction worldwide [6,7,8,9]. However, breast implants, as a foreign body, come with significant long-term drawbacks, including a reoperation rate of approximately 70% within 5–10 years. Reasons include implant displacement, rupture and/or visibility or rippling, breast animation, and capsular contracture that may also be associated with discomfort or pain [10], as well as the risk of developing breast implant illness and breast implant-associated anaplastic large-cell lymphoma [11,12]. Therefore, refinements of the IBBR approach are necessary. The use of specific implant surfaces as well as smaller implant volumes, in combination with non-vascularized fat as an adjunct autologous tissue to improve soft tissue coverage, might combine the durability of autologous reconstruction with the less invasive implant-based approach into a new hybrid technique.

Temporary reconstruction after a mastectomy with a tissue expander is often performed as a first step anyhow [13,14], to be later replaced by either a flap or an implant (i.e., expander-to-implant-based breast reconstruction; EBBR). During this intermediate phase, non-vascularized fat, harvested by means of liposuction, can be injected into the mastectomy flap to improve its thickness and tissue quality [15,16,17,18], especially when reconstruction with an implant is planned. Additionally, after completing the expansion process, the expander in place can be gradually deflated again while injecting the non-vascularized fat. This process may be repeated as needed, depending on the availability of fat and needs of the patient. The technical refinements of such a “reverse expansion” have lately been described by Stillaert et al. [19]. The reconstructive course can then be finalized by the explantation of the deflated expander and the insertion of a smaller breast implant to guarantee volume and foremost core projection, resulting in a hybrid, or composite reconstruction of the breast that is composed of non-vascularized adipose tissue and a breast implant [20]. This concept must be differentiated from the hybrid approach combining a microvascular flap with simultaneous implant placement for breast reconstruction, usually offered in patients with inadequate donor-site volume [21].

The use of autologous fat grafting has been widely investigated in both reconstructive and aesthetic procedures of the breast and proven to be safe [16,17,18,22]. Multi-stage composite breast reconstruction with autologous fat grafting aims to optimize soft tissue coverage and to allow a pre-pectoral implant placement, ideally prolonging the durability of the reconstruction and avoiding the drawbacks of submuscular placement, eventually resulting in revisional surgery [14,23,24,25,26,27,28]. Furthermore, the composite approach may help avoiding the use of synthetic meshes or acellular dermal matrices (ADMs), considering the fact that the thickened mastectomy flap provides not only better coverage but also better support. These adjunct materials are often used by plastic surgeons in breast reconstruction to give caudal and lateral support to the implant and eventually shape and contour the breast, particularly along the lower pole. This enables tension to be decreased on the distal part of the mastectomy flap while closing and aims to prevent wound dehiscence, implant extrusion, or aesthetic complications [29,30,31,32]. The reduced need for these devices due to a thicker mastectomy flap could thereby minimize associated costs and complications, such as increased risks of skin necrosis, seromas, or infections [23,32,33,34]. The main aim of the composite approach is, thus, to combine the advantages of autologous tissue and an implant to offer a durable and technically easier to perform type of breast reconstruction. Thus, it is not meant to replace flap-based breast reconstruction but aims to significantly improve the quality of IBBR [35,36,37,38,39].

The study’s aim was to analyze the clinical and radiological outcome of expander-to-implant breast reconstruction in combination with repeated fat grafting and reverse expansion.

## 2. Materials and Methods

### 2.1. Patients and Outcome Parameters

This prospective single center observational study included 25 consecutive patients, from August 2020 to April 2024, undergoing a bilateral mastectomy (nipple-sparing mastectomy (NSM), skin-sparing mastectomy (SSM), or skin-reducing mastectomy (SRM)) and immediate expander-to-implant multi-stage composite breast reconstruction using autologous fat grafting and reverse expansion. All surgeries were performed in the Department of Plastic, Reconstructive and Aesthetic Surgery of the Ente Ospedaliero Cantonale (EOC), in southern Switzerland. Patients undergoing radiotherapy, either unilaterally or bilaterally, were excluded from the study. Patient demographics, mastectomy data, details of the reconstructive procedure, and complications were recorded during the multi-stage procedure and at follow-up.

Subcutaneous thickness gain of the mastectomy flap of each breast was measured by comparing MRIs performed before the first session of autologous fat grafting and after the last session of autologous fat grafting with definitive implant placement. The MRI analyses were performed by two independent radiologists specialized in breast radiology (S.S., C.C.) and subsequently evaluated according to the number of fat grafting sessions and average volume of fat injected to finally be adjusted to the BMI. Complications that required surgery were defined as general complications. In addition, implant-related complications were assessed, including the evaluation of breast animation and the early grade of capsular contracture according to the Baker classification, later modified by Spear [40], as assessed by a senior plastic and reconstructive surgeon.

### 2.2. Multi-Stage Breast Reconstruction

Following a mastectomy performed by the breast surgeon, indocyanine green fluorescence was routinely executed to evaluate mastectomy flap perfusion. According to the resulting perfusion pattern, the mastectomy flap was trimmed as needed. The MRI-compatible tissue expander (Motiva Flora Tissue Expander, Establishment Labs, Alajuela, Costa Rica) [41] was filled, depending on the size of the remaining soft tissue, to avoid tension on the mastectomy flap. Thereafter, the expander was inserted in a pre-pectoral plane, fixed with strips of a partially absorbable mesh (ULTRAPRO^TM^ Macroporous, Johnson & Johnson, New Brunswick, NJ, USA) that were passed through the expander’s tabs as a figure of eight and sutured to the muscular fascia at the breast’s lower pole inframammary fold. The surgical access was closed using layered closure and absorbable sutures after a surgical drain was placed. After wound healing, the expander was gradually filled as needed with sterile saline until the desired breast volume was reached. Thereafter, the next surgical step was performed. Autologous fat was harvested (Body-jet, Human Med AG, Schwerin, Germany) using a modified Klein solution [42] depending on the donor sites available (paralumbar, buttock, abdomen, thigh). After sedimentation, the fat was injected in a multiplanar and multilayer fashion into the mastectomy flap between the deep dermal layer and the capsule adjacent to the expander, using a single-use injection canula. The expander was deflated as described by Stillaert et al. to accommodate the grafted fat [19]. These fat grafting procedures were performed as a standalone procedure on an out-patient basis. The procedure was repeated several times as needed. The last stage of the reconstruction included the explantation of the partially or completely deflated breast tissue expander, the injection of autologous fat, capsulotomies if needed, and the pre-pectoral insertion of the definitive implant (Motiva Ergonomix, Establishment Labs, Alajuela, Costa Rica; Polytech anatomical implants, Polytech Health & Aesthetics, Dieburg, Germany). In case of a mismatch of the mastectomy pocket and the definitive implant’s footprint, a synthetic non-absorbable mesh (TILOOP^®^ Bra Pocket, pfm medical GmbH, Cologne, Germany) was used to stabilize the implant to avoid lateral and/or caudal displacement.

### 2.3. Measurement of Tissue Thickness

**Figure 1:**
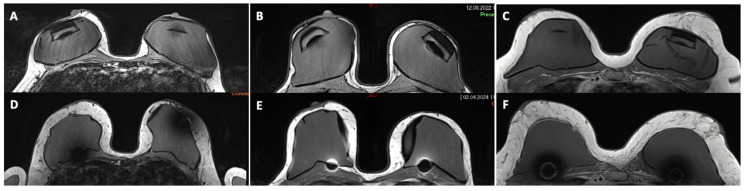
MRI examinations of a 37-year-old (**A**), 44-year-old (**B**), and 46-year-old (**C**) patient after a bilateral mastectomy sparing both skin and nipple–areolar complexes and pre-pectoral insertion of a breast tissue expander, before repeated sessions of autologous fat grafting of the mastectomy flap and reverse expansion of the expander. In (**D**–**F**), MRI examinations performed 3 months after 3 sessions of autologous fat grafting each and exchange of the expander with a definitive implant. Note the mean gain of tissue thickness in both breasts of 320% (**D**), 240% (**E**), and 120% (**F**).

All patients underwent a minimum of two MRI examinations to evaluate the subcutaneous tissue thickness before and after multi-stage hybrid reconstruction (Figure 1, Supplementary Materials Video S1). The first examination was performed once the tissue expander was fully inflated and before the first fat grafting session. The second examination was performed 3 months after the last stage of the reconstruction, including the last session of fat grafting. Breast MRI examinations were performed on a 3-T unit (MAGNETOM Skyra, Siemens Healthineers, Erlangen, Germany) with a dedicated eight-channel breast coil. The standard MRI protocol included axial turbo spin-echo T1-weighted Dixon sequences (TR 879 ms; TE 30 ms; flip angle 144°; slice thickness 3 mm), axial and sagittal T2-weighted turbo inversion recovery magnitude (TIRM) sequences (TR 4980 ms; TE 72 ms; flip angle 80°; slice thickness 3 mm), and axial DWI sequences (b values 0, 1000 s/mm^2^); TR 11,800 ms; TE 66 ms; flip angle 180°; slice thickness 3 mm).

On axial and sagittal sequences of the MR images, 4 specific and reproducible areas of the breast were defined (region of interest; ROI), starting from the nipple–areola complex (NAC) to the 4 cardinal points 2 cm cranial, medial, caudal, and lateral to the NAC, avoiding the expander and implant folds. For each ROI, the thickness of the subcutaneous tissue was measured in millimeters, as the maximum distance from the expander, or implant, to the skin. The value at time 0 of each corresponding ROI and breast was deducted from the corresponding value at time 1 to receive the gain in tissue thickness. Subcutaneous thickness measurements were independently performed by two board-certified breast radiologists (S.S.; C.C.). The final values used for analysis were derived by calculating the mean of both sets of measurements. Inter-reader reproducibility was assessed and showed no significant variability.

### 2.4. Statistics

All quantitative data are expressed as mean ± standard deviation (SD), with rounding to the second significant figure. Variations in subcutaneous tissue thickness were compared statistically using two-tailed Student’s *t*-test. Multiple comparisons were performed by 1-way analysis of variance (ANOVA) or 2-way ANOVA with repetition, depending on the groups’ equal size. Statistical significance was accepted for a *p*-value < 0.05. Statistical analysis was performed by using GraphPad Prism 10.0.0 (GraphPad Software, Boston, MA, USA).

## 3. Results

A total of 25 patients who completed bilateral multi-stage breast reconstruction were enrolled in the study, resulting in 50 reconstructed breasts. Patient demographics and characteristics regarding oncological treatment are summarized in Table 1. Among the reconstructed breasts, 24 (48%) underwent a therapeutic oncological mastectomy. Invasive ductal carcinoma was the most frequent tumor with 11 cases (22%), followed by in situ ductal carcinoma (5 breasts, 10%), lobular invasive carcinoma (3 breasts, 6%), and in situ lobular carcinoma (2 breasts, 4%). A prophylactic risk-reducing mastectomy of the contralateral breast was performed in 24 (48%) breasts. Six (12%) mastectomies were performed for gene mutation BRCA-, 7 (14%) for BRCA-2, as well as 11 (22%) for a “high-risk” profile of the patient (e.g., contralateral invasive tumor, family history, etc.) or due to the patient’s wishes. Four mastectomies (4%) were performed following extensive and painful fat necrosis after reduction mammaplasty or multiple painful nodules after silicone gel injection. NSM was performed in most of the cases (34 breasts), followed by SRM (pedicled nipple–areola complex in six cases, free nipple graft in four cases, and no nipple–areola complex preservation in two cases) and SSM (four breasts). The mean mastectomy weight was 320 ± 170 g. A total of 11 patients (44%) required additional oncological treatment with a combination of adjuvant hormonal therapy and immune therapy. Ten patients (40%) received at least one cycle of chemotherapy, of which four (16%) were administered in a neo-adjuvant regimen and six (24%) in an adjuvant regimen. None of the patients underwent adjuvant radiotherapy.

Details regarding multi-stage breast reconstruction are provided in Table 2. Mean BMI at the time of the mastectomy was 23 ± 4 kg/m^2^ and did not change significantly compared to BMI at the time of the first and last fat grafting session (24 ± 4 kg/m^2^ vs. 24 ± 4 kg/m^2^, respectively), indicating a stable weight throughout the reconstructive treatment (Table 1).

Following expander placement, three patients (six breasts, 12%) underwent only one session of fat grafting, administered during the replacement of the expander with a definitive implant, whereas 44 breasts (88%) underwent two or more fat grafting sessions. Patients underwent 2.5 ± 0.6 fat grafting sessions on average, with a mean volume of injected fat of 170 ± 60 mL per breast and per session.

From the time of the mastectomy with the insertion of the expander to its exchange for the final implant, a total of 244 injections of the 50 expanders were made, either to fill before initiating fat grafting (*n* = 170) or to deflate during reversed expansion (*n* = 74) the expander, resulting in a mean of 4.9 injections per expander. 

Ergonomic implants were preferred over anatomically shaped implants in most cases (76% vs. 24%). A synthetic, non-absorbable mesh was used additionally while placing the nanotextured implant in 12 breasts (24%), to prevent lateral and/or caudal dislocation. The average duration of the total reconstructive process from the mastectomy to final implant insertion lasted 12 ± 5 months, with mean intervals between the individual fat grafting sessions of 4.5 ± 1.2 months. The mean follow-up time at the end of the reconstructive process was 17 ± 8 months. For the six patients who required adjuvant chemotherapy, the reconstructive process was significantly longer than the overall average, due to a mean time between the mastectomy and the first fat grafting session of 9.3 ± 5.0 months and an average duration of the reconstructive process of 15 ± 5 months.

Complications related to breast reconstruction requiring surgery occurred in eight breasts (16%; see Table 3). Most of these complications occurred during the reconstructive process, with iatrogenic expander puncture being the most frequent one (three cases, 6%). The second most frequent complication was an infection of the skin pocket following two mastectomies (4%), likely due to the limited early perfusion of the mastectomy flap and delayed wound healing. Both infections were effectively managed with early surgical revision, including surgical debridement, pocket irrigation, and replacement of the expander and systemic antibiotic therapy. Following the mastectomy and the temporary reconstruction with expander, we observed one postoperative hematoma (2%), which required surgical evacuation. One patient each underwent additional fat grafting following completed reconstruction due to volume asymmetry and peri-areolar scar correction for unaesthetic scar formation (4%). It is noteworthy that one patient underwent surgical radicalization due to microscopical non-radical oncological surgery (R1) right after the mastectomy. In this study, so far, only one complication (2%) was directly associated with fat grafting, a painful nodule that was initially detected clinically, then analyzed with ultrasound and subsequently biopsied, revealing fat necrosis following multiple fat grafting sessions. No seromas requiring puncture or surgical interventions were found. During follow-up, no symptomatic grade III and IV capsular contractures were observed. Instead, 96% and 4% of all patients presented with a grade Ia or Ib, respectively, grade II capsular contracture (Table 3). Furthermore, one case of bilateral implant rippling was observed in the upper inner quadrants of the breasts in a rather thin patient (BMI between 19 and 20 kg/m^2^ from mastectomy to final implant placement). Finally, it is noteworthy that no case of breast animation was seen (Supplementary Materials Video S2).

**Figure 2:**
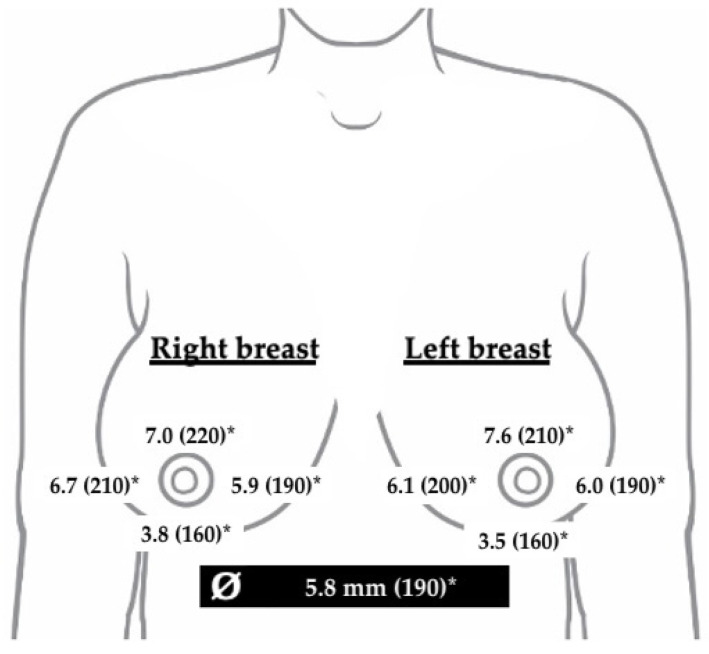
Mean subcutaneous tissue in mm and in (%) compared to baseline values (100%), following repeated autologous fat grafting in multi-stage composite breast reconstruction. Tissue thickness is shown for each region of interests (4 breast quadrants) and per breast. * *p*-value < 0.05 vs. baseline thickness (after mastectomy).

The mean gain in subcutaneous tissue thickness of all four quadrants of the breasts was 5.8 ± 3.5 mm (92%). These mean values showed that overall mastectomy flap thickness almost doubled following repeated fat grafting when compared to baseline values before fat grafting. The increase was markedly higher in the upper quadrants (7.3 ± 5.4 mm; 110%) when compared to the remaining ROIs, particularly the lower quadrants (3.7 ± 3.8 mm, 58%). No statistically significant differences were observed between the right and the left breast (Figure 2).

**Figure 3:**
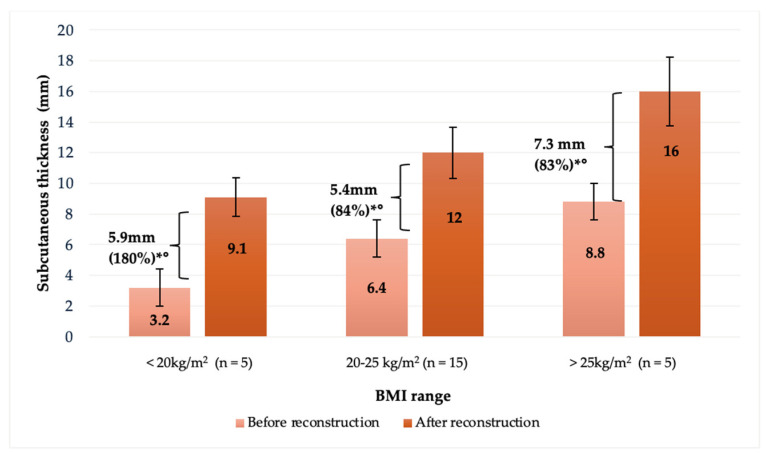
Evolution of gain in subcutaneous tissue thickness during repeated fat grafting in multi-stage breast reconstruction according to BMI (underweight: <20kg/m^2^; normal weight: 20–25 kg/m^2^; overweight: >25 kg/m^2^). Error bars illustrate standard deviation (SD). * *p*-value < 0.05 vs. baseline thickness (after mastectomy and finalized expansion). No statistical correlation was found for mean gain (in mm) across the three groups (° *p*-value > 0.05).

Subcutaneous tissue thickness before fat grating correlated with the patients’ baseline BMI, i.e., the lower the BMI, the thinner the subcutaneous thickness (Figure 3). Underweight patients (BMI < 20 kg/m^2^) underwent an average of 2.2 ± 1.0 fat grafting session, with a mean of 110 ± 40 mL of fat injected per session, resulting in a mean increase in subcutaneous thickness of 5.9 ± 1.4 mm (180%), whereas normal-weight patients (BMI 20–25 kg/m^2^) underwent a mean of 2.3 ± 0.7 fat grafting sessions with a mean of 170 ± 60 mL of fat injected per session, leading to a 5.4 ± 3.8 mm (84%) increase in thickness. Overweight patients in contrast (BMI > 25 kg/m^2^) received an average of 2.8 ± 0.4 fat grafting sessions, with a mean of 190 ± 50 mL of fat injected per session, resulting in a 7.3 ± 3.1 mm (83%) increase in tissue thickness (Figure 3). In all three groups, the gain of subcutaneous thickness was statistically significant when compared to baseline tissue thickness, but although thicker patients underwent more fat grafting sessions and had a greater volume of fat injected, no statistical difference in thickness gain was observed among the three groups.

**Figure 4:**
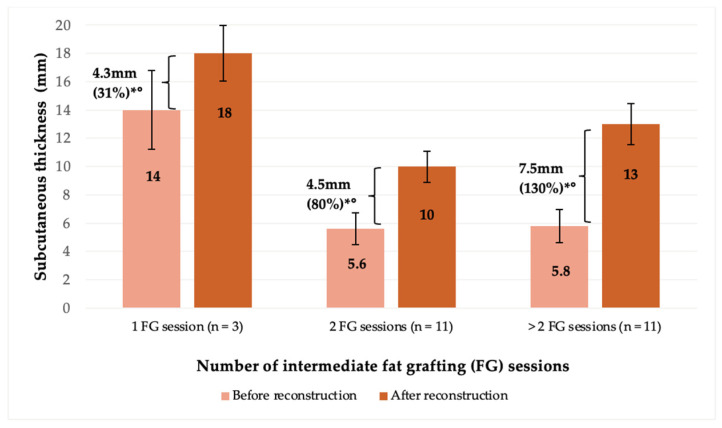
Subcutaneous tissue thickness gain according to fat grafting sessions. Evolution of subcutaneous tissue thickness during repeated fat grafting in multi-stage breast reconstruction according to the number of fat grafting sessions. Error bars illustrate standard deviation (SD). * *p*-value < 0.05 vs. baseline thickness (after mastectomy and finalized expansion). Statistical correlation was found for mean gain (in mm) across the three groups (° *p*-value < 0.05).

An increase in subcutaneous tissue thickness was also analyzed according to the number of fat grafting sessions the patients underwent. Patients who underwent one fat grafting session were injected with an average of 190 ± 110 mL of fat per session, resulting in a 4.3 ± 3.4 mm (31%) increase in thickness. Patients who underwent two fat grafting sessions and three fat grafting sessions were injected a mean of 150 ± 60 mL and 170 ± 50 mL of fat per breast, respectively, resulting in a 4.5 ± 2.8 mm (80%) and 7.5 ± 3.0 mm (130%) gain in subcutaneous thickness, respectively (Figure 4). For all examined groups, this gain in subcutaneous thickness of the mastectomy flap was statistically significant compared to baseline tissue thickness. Additionally, a statistical correlation between the number of fat grafting sessions and an increase in tissue thickness was found across all groups.

## 4. Discussion

This study highlights the efficacy of the repeated use of autologous fat grafting in expander-to-implant breast reconstruction. In all 25 patients, a significant increase in soft tissue thickness has been achieved for better implant coverage, thus allowing the positioning of the implant in the pre-pectoral plane. The additional use of a synthetic mesh was only necessary in the presence of a footprint mismatch of the larger expander and the smaller definitive implant, concerning only a minority of the cases. It is our opinion that the use of the pre-pectoral plane—whenever possible—helps avoid complications frequently associated with submuscular implant placement, such as breast animation or displacement, that ultimately disturb the patient and may require revisional surgery [14,23,24,25,26,27]. Moreover, the initial use of the pre-pectoral plane leaves the submuscular plane untouched for the possible case of an implant change over the course of the patient’s remaining life span with the option to change the implant’s pocket to an untouched submuscular plane.

The use of autologous fat grafting as a standalone procedure into the mastectomy flap was associated with a significant increase in subcutaneous thickness in all breast regions. A greater increase was observed in the upper quadrants followed by the outer quadrants, while the lower quadrants showed the smallest gain (Figure 2). Importantly, no differences were observed in the baseline subcutaneous thickness between the quadrants prior to fat grafting, with a relatively uniform tissue envelope. This suggests that the variations in tissue gain between the four quadrants were a direct result of the grafting procedure rather than pre-existing differences in tissue thickness. One hypothesis is that the weight and pressure exerted by the tissue expander or implant might compress the subcutaneous tissue in lower quadrants. This may also be caused by the fact that both the expander and definitive implant do not adhere to the surrounding tissues due to their rather smooth surface. Conversely, the lateral and foremost upper quadrants, being under less strain, might provide a more favorable environment for fat cell survival and integration. This differential strain hypothesis aligns with the findings of other studies that suggest better graft take rates in areas with less mechanical stress and more favorable vascularity [19,22].

**Figure 5:**
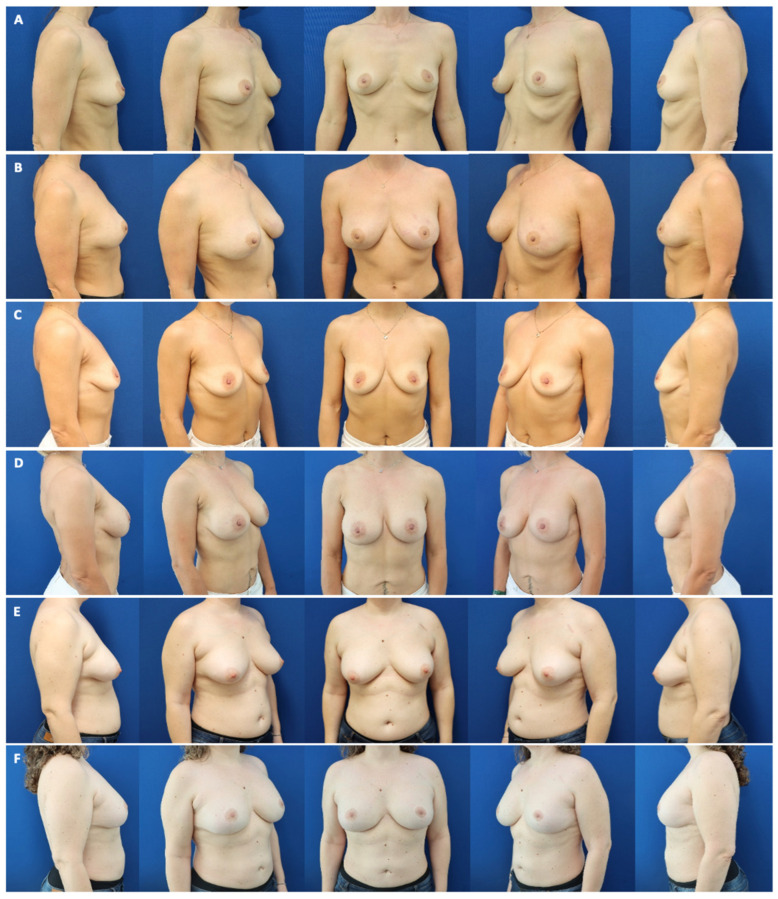
(**A**) A 37-year-old underweight patient with a BMI of 19 kg/m^2^ and invasive ductal carcinoma of the left breast and desire of mastectomy of the right breast before bilateral nipple-sparing mastectomy with inframammary approach (mastectomy weight ca. 150 g each) and pre-pectoral expander placement (Flora XMF-54; Establishment Labs). (**B**) One year following three sessions of autologous fat grafting (170 mL, 100 mL, and 160 mL each), and reverse expansion and exchange of the expanders for definitive, ergonomic implants (Motiva E2SM 250 mL, mini projection; Establishment Labs). BMI after finalization of reconstruction: 21 kg/m^2^. (**C**) A 44-year-old normal-weight patient with a BMI of 21 kg/m^2^ and BRCA-1 gene mutation before bilateral nipple-sparing mastectomy with inframammary approach (mastectomy weight ca. 220 g each) and pre-pectoral expander placement (Flora XMF-54; Establishment Labs). (**D**) Nine months following three sessions of autologous fat grafting (150 mL, 110 mL, and 120 mL each), and reverse expansion and exchange of the expanders for definitive, ergonomic implants (Motiva E2SM 330 mL, mini projection; Establishment Labs). BMI after finalization of reconstruction: 23 kg/m^2^. (**E**) A 46-year-old overweight patient with a BMI of 30 kg/m^2^, invasive ductal carcinoma T2N0, G3 right breast, and BRCA-1 gene mutation before bilateral skin-reducing mastectomy and pedicled nipple–areola complex (mastectomy weight ca. 470 g each), and pre-pectoral expander placement (Flora XMM-66; Establishment Labs). (**F**) Eighteen months following three sessions of autologous fat grafting (250 mL, 220 mL, and 190 mL each), and reverse expansion and exchange of the expanders for definitive, ergonomic implants (Motiva E2SD 360 mL; demi projection, Establishment Labs). BMI after finalization of reconstruction: 29 kg/m^2^.

The correlation between the number of fat grafting sessions and increased subcutaneous thickness observed in this study underlines the importance of optimizing the fat grafting regimen. Patients undergoing more fat grafting sessions experienced a higher increase in tissue thickness. This can be attributed to the ability of the injected fat to integrate into the surrounding tissue, creating a stable, vascularized environment that supports further fat grafting. Currently, we have not observed any plateau effect regarding a gain in mastectomy flap thickness after a mean number of 2.5 fat grafting sessions per patient. Thus, it could be assumed that additional fat grafting sessions would further increase subcutaneous mastectomy flap thickness [43]. Since each fat grafting session provides a consistent and predictable benefit, patients can undergo several sessions to achieve their desired results without concerns about diminishing efficacy. These results also show that fat grafting was effective across all three BMI ranges examined in the present study (Figure 3 and Figure 5). It is, however, interesting to see that patients with a lower BMI tended to achieve a higher percentage increase in thickness with fewer sessions and lower volumes of autologous fat injected when compared to those with a higher BMI. This is likely due to the thinner baseline subcutaneous tissue thickness in lower BMI patients, where even small volumes of injected fat can result in proportionally larger increases in thickness. For patients with a higher BMI, a more intensive fat grafting regimen might be required to achieve comparable results. This variability in response underscores the importance of tailoring the fat grafting regimen to individual patient characteristics. However, the effectiveness of the technique across all BMI categories, i.e., underweight, normal weight, and overweight, demonstrates the flexibility and adaptability of this surgical technique as a reliable option for a diverse patient population.

The aesthetic outcomes of the used composite technique were favorable, with most patients displaying a very natural-looking breast. No cases of symptomatic capsular contracture (grade III–IV) were observed and there was only one case of mild capsular contracture (grade II). Although the mean follow-up period of the currently presented cohort is only approximately 17 months, this is a promising initial result compared to many other series of IBBR, where capsular contracture represents the most common complication and the major cause of surgical revision [44]. A recent meta-analysis showed no significant difference in the incidence of capsular contracture when comparing subpectoral and pre-pectoral implant placements [45]. Furthermore, there is evidence that enhancement of peri-prosthetic tissue with fat grafting decreases the collagen content, density, and fiber alignment of capsular tissue surrounding implants [46]. This might be one of the reasons that autologous fat grafting provides therapeutic benefits in cases of symptomatic capsular contracture by disrupting collagen fiber alignment and decreasing capsular thickness [47].

Despite its benefits, the multi-stage composite approach is not without challenges. Most complications observed in this study were directly related to the tissue expander, such as iatrogenic expander puncture. Hematoma and infection, on the other hand, should be seen as complications of the mastectomy itself, as well as delayed healing of the mastectomy flap. Notably, these complications are not unique to the composite technique but rather inherent risks of a staged breast reconstruction process [13,48,49,50]. Of interest, only one complication was directly related to autologous fat grafting. Following a total of 118 fat grafting sessions, one diagnostic percutaneous biopsy of a painful nodule was performed that resulted in being fat necrosis. This finding supports the growing body of evidence that autologous post-surgical fat grafting is a safe and effective technique for breast reconstruction [10,22]. However, it is important to acknowledge the potential long-term risks associated with fat grafting, particularly fat necrosis, which may manifest as palpable and sometimes painful nodules, but especially as microcalcifications [51,52]. This fact can impede postoperative imaging and eventually result in more frequent ultrasound examinations or even biopsies to rule out malignancy [16,17,51,52]. Despite oncological safety after autologous fat grafting to the breast already being shown and reliable classification having been described to differentiate between benign lesions and malignant lesions [53], a more thorough postoperative imaging remains a major consideration in oncological patients.

Another important factor to consider when offering multi-stage composite breast reconstruction, is the need for repeated surgeries and the overall longer duration of the reconstructive process. While according to consensual recommendation and the current literature [16,54,55], fat grafting sessions could be performed about every 3 months in an ideal setting, the mean interval between the surgical steps was slightly longer (4.5 ± 1.2 months), resulting in a prolonged overall duration to achieve complete breast reconstruction of approximately one year (12 ± 5 months). This prolonged time can be attributed to various factors, including the need for adjuvant chemotherapy but also patient scheduling and individual preferences. This total time of approximately 1 year may discourage some patients from completing the necessary fat grafting sessions, potentially leading to suboptimal results in gain of subcutaneous tissue thickness and, eventually, implant coverage. This is particularly relevant, given that the effectiveness of this multi-stage composite technique appears to be somewhat “dose-dependent”, i.e., more fat grafting sessions result in an overall thicker mastectomy flap and ultimately better aesthetic outcome.

In traditional expander-to-implant BR, autologous fat grafting is often performed during a third surgery, following the final implant placement, to refine the aesthetic appearance of the reconstructed breast [56,57,58]. These additional procedures typically address some asymmetries, contour irregularities, and implant visibility. Considering the present data, it is tempting to speculate that hybrid reconstruction can reduce the need for this type of refinement, as adjustments regarding slight volume asymmetries may already be addressed during the reverse expansion process. In fact, in the presented cohort, only one patient (4%) required additional fat grafting to correct volume asymmetry after the placement of the definitive implant. Although this multi-stage composite approach requires a longer duration of reconstruction, we believe that more durable implant-based breast reconstructions with fewer surgical revisions in the long run and increased patient satisfaction can be achieved.

### Limitations

The present study has certain limitations. Most importantly, the sample size is relatively small, with only 25 consecutive patients and a total of 50 reconstructed breasts. While we think that 50 breasts are sufficient to provide an early evaluation of this emerging technique and to present a proof of concept, a larger cohort will be needed to validate these results and ensure their relevancy to a broader patient population over time. Additionally, the follow-up period is still short, with an average of approximately 17 months. This limits the ability to assess long-term outcomes such as the durability of the reconstructive result that will mainly be conditioned by the thickness of the subcutaneous tissue covering the implant. Over time, increased subcutaneous tissue thickness resulting from well-integrated fat will rather underlie significant weight changes of the patient than resorption after years [59,60,61,62]. Therefore, future studies with longer follow-up will be essential to determine the reliability and durability of this reconstructive approach, including the rate of long-term implant-based complications. Moreover, the lack of a control group makes it difficult to fully isolate the effects of the multi-stage composite approach. This is particularly relevant when addressing specific aspects of the procedure, such as the overall duration, the use of synthetic meshes, or the associated complications. However, despite the absence of a control group, the preliminary results of the current series are very promising and show a total rate of complications during the reconstructive process of 22%. The reoperation rate after completed multi-stage reconstruction at a mean follow-up time of 17 months is 4% and the rate of implant loss 0% so far. In recent studies, Wilkins et al. [63] and Bennet et al. [64] described an overall rate of complications for implant-based BR of 25% and 31%, respectively. The reoperation rate at 1 year and 2 year follow-up was 18% and 19%, respectively, and the rate of reconstructive failure for implant removal 6% and 7%, respectively.

## 5. Conclusions

The multi-stage composite EBBR that foresees repeated autologous fat grafting sessions offers a promising alternative to standard IBBR. The present study demonstrates that this approach is associated with a significant gain in tissue thickness in all representative areas of the mastectomy flap. Moreover, the increase in tissue thickness correlates well with the numbers of fat grafting sessions, regardless of the patient’s BMI. While the findings suggest that optimizing soft tissue coverage with multiple fat grafting sessions may enable a more frequent use of the pre-pectoral plane, if possible without the need for biologic or synthetic meshes, the small sample size warrants caution in generalizing these results. However, the use of small implants continues to ensure a central mound and core projection of the reconstructed breast, and the improved implant coverage appears to achieve a more natural appearance of the reconstructed breast and may contribute to implant durability over time.

## Figures and Tables

**Table 1:** Patient demographics.

Clinical Demographic Data	Values
**No. of patients**	25
**No. of breasts (Ref.)**	50
**Diagnosis**	
IDC/ILC: *n* (%)	14, t11/3 (28)
DCIS/LCIS: *n* (%)	8 (16)
Risk reducing (BRCA-1, BRCA-2, high risk): *n* (%)	24–6/7/11 (48)
Others (siliconome, fat necrosis): *n* (%)	4 (8)
**Mean age at mastectomy ± SD: (yr)**	48 ± 8
**Mastectomy type: *n* (%)**	
NSM	34 (68)
SSM	4 (8)
SRM (with NAC preservation/without NAC preservation)	12–6/6 (24)
**Mean mastectomy weight ± SD: (g)**	320 ± 170
**Mean BMI ± SD: (kg/m^2^)**	
At mastectomy	23 ± 4
Before first session of FG	24 ± 4
After final reconstruction	24 ± 4

IDC/ILC, invasive ductal/lobular carcinoma; DCIS/LCIS, ductal/lobular carcinoma in situ; BRCA-1 and -2, Breast Cancer Susceptibility Gene 1 and 2; SD, standard deviation; NSM, nipple-sparing mastectomy; SSM, skin-sparing mastectomy, SRM, skin-reducing mastectomy; NAC, nipple–areola complex; BMI, body mass index; FG, fat grafting.

**Table 2:** Treatment details.

Treatment-Related Data	Values
**Additional treatment: *n* patients (%)**	
Hormonal therapy/immunotherapy	11 (44)
Chemotherapy	10 (40)
Adjuvant	6 (24)
Neo-adjuvant	4 (16)
**Reverse expansion, i.e., >1 fat grafting session: *n* patients (%)**	22 (88)
**Mean number of FG sessions ± SD: n**	2.5 ± 0.6
**Mean volume of fat injected per breast ± SD (mL)**	170 ± 60
**Type of final implant**	
Round ergonomic (Motiva, Establishment Labs): *n* (%)	38 (76)
Anatomical (Polytech Health & Aesthetics): *n* (%)	12 (24)
**Mean volume of final implant ± SD: (mL)**	360 ± 90
**Mean time between surgical stages ± SD: (months)**	
Mastectomy to first AFG session	5.8 ± 3.3
First to second AFG session	4.1 ± 1.5
Second to third AFG session	5.2 ± 3.0
Third to fourth AFG session	3.0 ± 0.0
**Mean duration of reconstructive procedure ± SD: (months)**	12 ± 5
**Mean follow-up time ± SD: (months)**	17 ± 8
**Use of synthetic mesh: *n* (%)**	
None	38 (76)
Inner bra (TILOOP Bra Pocket, pfm medical)	12 (24)

FG, fat grafting; SD, standard deviation.

**Table 3:** Complications.

Type of Complication	Values *n* (%)
**General complications requiring surgical intervention**	8 (16)
Hematoma	1 (2)
Infection	2 (4)
Expander rupture	3 (6)
Seroma	0 (0)
Volume asymmetry	1 (2)
Unaesthetic scar	1 (2)
**Implant-related complications (two breasts required surgical revision)**	
Breast animation	0 (0)
Rippling	2 (4)
Grade of capsular contracture	
IA	38 (76)
IB	10 (20)
II	2 (4)
III	0 (0)
IV	0 (0)
**Fat grafting-related complication (one breast required biopsy)**	
Painful nodule of fat necrosis	1 (2)

Overview of general and implant-related complications. Capsular contracture is defined as described by the modified Baker–Spear classification [40].

## Data Availability

The data presented in this study are available on request from the corresponding author due to privacy and ethical restrictions.

## Supplementary Materials

The following supporting information can be downloaded at: https://www.mdpi.com/article/10.3390/jcm14020337/s1. Video S1: MRI sequences of a 37-year-old (A), 44-year-old (B), and 46-year-old (C) patient after bilateral mastectomy, sparing both the skin and nipple–areolar complexes, with pre-pectoral insertion of a breast tissue expander. These sequences were captured before repeated sessions of autologous fat grafting to the mastectomy flap and reverse expansion of the expander. In (D–F), the corresponding MRI sequences were performed 3 months after 3 sessions of autologous fat grafting each and the exchange of the expander with a definitive implant. Video S2: Repeated contraction of bilateral pectoralis major muscles in a 37-year-old (A), 44-year-old (B), and 46-year-old (C) patient after bilateral mastectomy sparing both the skin and nipple–areolar complexes, with pre-pectoral expander-to-implant breast reconstruction and repeated autologous fat grafting sessions while gradually deflating the expander. Note the almost complete absence of breast animation, even in the 44-year-old patient having the implant stabilized with a synthetic, non-absorbable mesh to prevent caudal “bottoming out” and lateral migration.
